# Mildew-Induced Resistance in Roses Against *Spodoptera exigua* and Chemical Compounds Produced During the Defense Response

**DOI:** 10.3390/molecules31111955

**Published:** 2026-06-04

**Authors:** Guiyan Chen, Ruiqing Jiang, Jun Feng, Guolei Zhu, Bin Yang, Fazhong Yang, Yunxian Li

**Affiliations:** 1 Key Laboratory of State Forestry and Grassland Administration on Highly-Efficient Utilization of Forestry Biomass Resources in Southwest China, Southwest Forestry University, Kunming 650224, China; 2 Kunming Natural Resources Comprehensive Investigation Center, China Geological Survey, Kunming 650100, China; 3 Pu’er University, Pu’er 665000, China

**Keywords:** *Podosphaera pannosa*, *Rosa chinensis*, *Spodoptera exigua*, LC-MS, EC50, plant-mediated interaction, chemical mechanisms

## Abstract

In the ternary system of *Podosphaera pannosa*, *Spodoptera exigua*, and *Rosa chinensis*, volatiles from infected roses suppress *S. exigua* oviposition, but the non-volatile-mediated resistance mechanism remains unclear. This study investigated mildew-induced resistance in roses by assessing larval feeding responses to leaf extracts. Infected rose leaf extracts significantly inhibited larval feeding (68.55% inhibition; *t* = 4.742, d*f* = 28, *p* = 0.0002). Untargeted LC-MS metabolomics identified 1046 metabolites in *R*. *chinensis*. *P. pannosa* infection altered the rose metabolome, yielding 64 differentially accumulated metabolites (47 up, 17 down). Among these, five compounds were markedly upregulated: dehydro(11,12) ursolic acid lactone (**A**), maslinic acid (**B**), trametenolic acid B (**C**), betulonic acid (**D**), and ganolucidic acid B (**E**). All five inhibited larval feeding. Compound A showed the strongest activity (80.99% at 0.1 mg/mL), with an EC_50_ of 0.015 mg/mL. EC_50_ values for compounds **B**–**E** were 1.256, 0.067, 1.189, and 0.014 mg/mL, respectively. This study elucidates a resistance mechanism of mildew-induced roses and suggests that compounds with low EC_50_ values (e.g., **A** and **E**) warrant further investigation as potential candidates for eco-friendly pest management under laboratory conditions.

## 1. Introduction

In natural ecosystems, plants are often simultaneously exposed to dual stresses caused by pathogen infection and herbivorous insect feeding. The tripartite interactions among these components are complex and can manifest either as synergistic effects that exacerbate plant damage or as antagonistic effects mediated by the host plant [1]. Induced plant defense responses triggered by biotic agents, including both pathogens and beneficial microorganisms, are known to enhance resistance to insect herbivores across diverse plant species. For instance, inoculation of sweet basil with plant growth-promoting rhizobacteria (PGPR) significantly enhanced both biochemical and structural defenses [2]. Bertasello et al. [3] demonstrated that infection of sugarcane by Sugarcane Yellow Leaf Virus significantly altered the feeding behavior and biological performance of the aphid vector *Melanaphis sacchari*, illustrating how pathogen infection shapes plant–insect interactions in tripartite systems. Extending this framework, a recent study on arbuscular mycorrhizal fungi revealed that beneficial root-associated fungi alter the phyllosphere microbial community and fine-tune plant defenses, thereby protecting the host against co-infliction by both pea aphids and pathogens [4]. Complementing these plant-associated systems, a recent study on ambrosia beetles also documented a fungus-mediated ternary interaction, where symbiotic fungal volatile organic compounds (VOCs) drive host selection and dispersal behavior in *Megaplatypus mutatus* [5]. While some ecological studies have identified certain patterns of the interactions in the ternary system, such as the inhibitory effect of powdery mildew (*Podosphaera pannosa*) infection on the ovipositional behavior of *Spodoptera exigua* on roses [6], the role of non-volatile plant secondary metabolites in this resistance remains unknown. Most previous studies have focused on VOCs as mediators of plant–pathogen–insect interactions [7,8]. However, the role of non-volatile secondary metabolites—especially triterpenoids—in mildew-induced resistance to *S. exigua* remains uncharacterized. Elucidating the underlying mechanisms of these plant–pathogen–insect interactions will advance our understanding of multi-species relationships in ecosystems and provide a theoretical basis for sustainable pest management. Given the growing challenges of pesticide resistance, addressing this knowledge gap is critical for modern plant protection. *S. exigua* larvae furthermore exhibit clear feeding preferences, with consumption rates rising over time on preferred hosts [9].

Cut roses remain the top-selling flower among the four major cut flowers worldwide, accounting for 34% of global auction volume, and are regarded as the most economically valuable ornamental plants due to their high diversity of fragrances and colors [10,11]. In previous studies, our research group used rose leaves as experimental material to investigate plant defense responses to pathogen infection and herbivore feeding [7,8]. However, the prevalence of diseases and pests has severely impacted rose yield and economic viability. Taking Yunnan Province as an example, due to suboptimal greenhouse cultivation conditions, the incidence of *P. pannosa* and the infestation rate of *S. exigua* are particularly prominent [12]. *P. pannosa* is a fungal pathogen that exclusively infects rose plants, causing damage to leaves, petioles, branches, and buds, thereby compromising both growth and ornamental value [13]. Diseases affecting rose production, including powdery mildew caused by *Podosphaera pannosa*, as well as other viral, bacterial, and fungal pathogens, spread rapidly and widely, complicating prevention and control efforts and causing significant economic losses in agriculture. Meanwhile, *S. exigua* is a typical polyphagous herbivorous pest that damages a broad range of cultivated crops. Its host plants include, but are not limited to, cotton, tomato, celery, lettuce, cabbage, and rose, further exacerbating the challenges of pest management in diversified agricultural ecosystems [14]. To date, the control of *S. exigua* still relies mainly on chemical pesticides, including chlorinated hydrocarbons, organophosphates, carbamates, and pyrethroids [15]. Although insecticides are effective in managing insect pests and diseases in the short term, their prolonged use can result in the development of resistance in *S. exigua* and *P. pannosa* [16]. As a result, effective pest control has emerged as a critical challenge that must be addressed to advance the rose industry.

The long-term overuse of synthetic pesticides has caused ecological risks and health concerns, prompting the search for eco-friendly alternatives [17]. Botanical compounds, with diverse structures and bioactivities, have emerged as promising crop protection agents, helping plants resist pests, diseases, and abiotic stress while attracting pollinators. Unlike synthetic pesticides, plant-derived products are generally safer and environmentally benign, supporting the development of novel biopesticides [18]. Over 230 plant extracts exhibit antifeedant and repellent activities [11,19]. However, their practical application requires formulation optimization [20]. For example, neem (*Azadirachta indica*)-based products exhibit broad-spectrum insecticidal activity against multiple arthropod pests, including mosquito vectors of public health importance, with eco-friendly features and multiple mechanisms of action [21]. Similarly, extracts of A. indica and Nicotiana tabacum show toxicity against *Plutella xylostella* [22]. Within the ternary system comprising *P. pannosa*, *R. chinensis* and *S. exigua*, infection of roses by *P. pannosa* was found to alter both the volatile and non-volatile compounds of rose plants [7]. These alterations have been shown to detrimentally affect the host selection behavior of *S. exigua* moths, although the precise chemical mechanism responsible for this effect remains unknown. In the present research, we focused on demonstrating the changes in secondary metabolites in *P. pannosa*-infected rose plants and identifying the biologically active chemical constituents. Ultimately, we aimed to elucidate the chemical resistance mechanisms and provide viable products for developing eco-friendly, efficient, and sustainable control methods for *S. exigua*.

## 2. Results

### 2.1. Effect of Extracts on the Feeding of S. exigua Larvae

**Figure 1:**
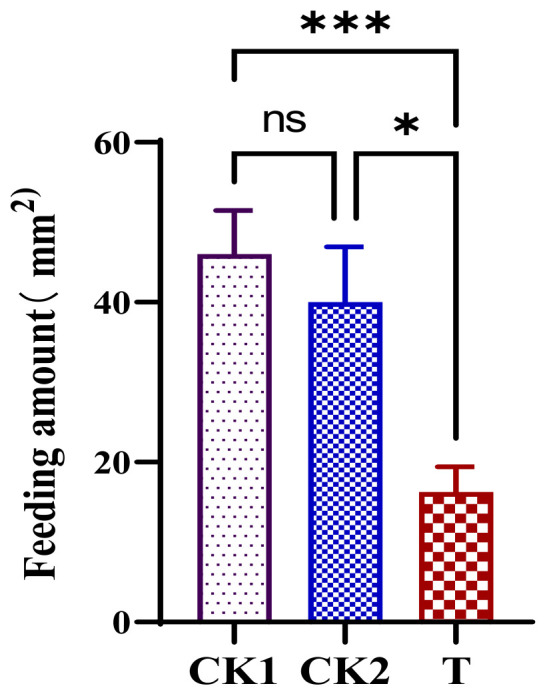
The effect of rose extract on the feeding behavior of *S. exigua*. CK1: rose leaves from healthy plants; CK2: rose leaves treated with a 50% methanol aqueous solution; and T: rose leaves infected with *P. pannosa* (* *p* < 0.05; *** *p* < 0.001).

In the experiment, compared to leaves treated with either 5 mg/mL extract from healthy roses (CK1) or the 50% methanol aqueous solution (CK2), *S. exigua* larvae consumed significantly less leaf tissue from leaves treated with extract derived from *P. pannosa*-infected roses (T) after 24 h (Figure 1). Specifically, treatment T reduced larval feeding by 69% relative to CK1 (*t* = 4.742, d*f* = 28, *p* = 0.0002); in comparison to CK2, treatment T extracted from *P. pannosa*-infected roses markedly suppressed the feeding activity of beet armyworm larvae, resulting in an antifeedant rate of 41% (*t* = 2.820; d*f* = 28; *p* = 0.009). While there was no significant difference between CK1 and CK2 (*t* = 1.384, d*f* = 28, *p* = 0.809), the results indicated that the 50% methanol solution had no antifeedant effect. Consequently, solvent interference in the three-choice bioassay was ruled out, confirming that extracts from the mildewed roses possess an antifeedant effect on *S. exigua*.

### 2.2. Influence of Mildew on Rose Metabolomics

**Figure 2:**
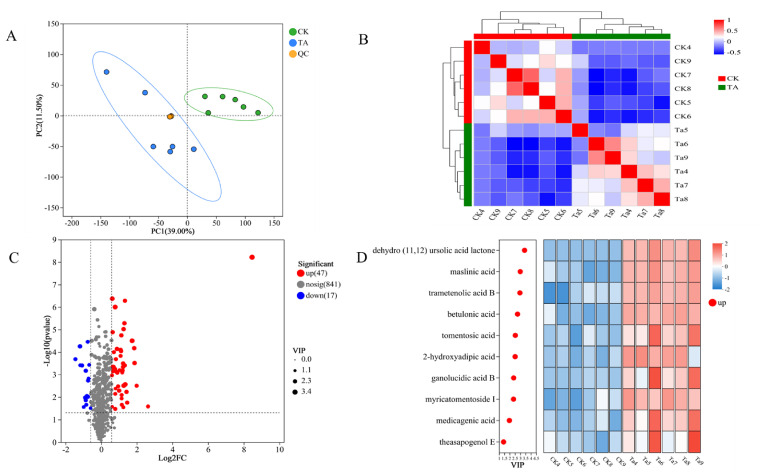
Effects of *P. pannosa* infection on metabolomic profiles. (**A**) Principal component analysis (PCA) between the samples (QC indicates data quality control; healthy (CK) and mildew-infected (Ta)). The ellipses indicate the range at a 95% confidence level. (**B**) Heatmap of correlation in positive and negative ion modes. The relative size of the correlation coefficient between the samples is reflected by the color; the left and upper sides of the figure are the clustering results. (**C**) Volcano plots of DAMs in Ta vs. CK: “up” means upregulated, “down” means downregulated, and “nosig” means not significant. (**D**) A VIP bubble plot of metabolites, where the *Y*-axis represents metabolites, and the *X*-axis represents VIP values.

To investigate the chemical mechanism underlying *P. pannosa*-induced resistance in roses against *S. exigua*, a metabolomic analysis was performed on 12 rose samples, comprising 6 healthy samples (CK4–CK9) and 6 *P. pannosa*-infected samples (Ta4–Ta9). Using liquid chromatography–mass spectrometry (LC-MS), a total of 1046 metabolites were detected. Principal component analysis (PCA, Figure 2A) revealed that, upon *P. pannosa* infection, significant alterations occurred in the rose metabolomic profile. The confidence intervals of the healthy control and Ta were distinctly separated, while QC samples consistently clustered within the confidence interval of the Ta group, thereby confirming the reliability of the analytical method. Moreover, hierarchical clustering heatmap analysis (Figure 2B) demonstrated that all six healthy control samples (CK4–CK9) formed a distinct cluster, showing little similarity to the Ta group. These findings collectively indicated that *P. pannosa* infection significantly affects both the expression levels and the compositional structure of rose metabolites.

**Figure 3:**
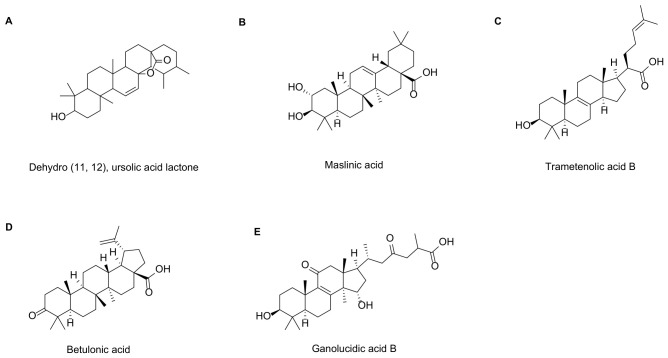
Names and structures of the five upregulated compounds. (**A**) Dehydro(11,12), ursolic acid lactone; (**B**) Maslinic acid; (**C**) Trametenolic acid B; (**D**) Betulonic acid; (**E**) Ganolucidic acid B.

DAMs were screened using analysis software, including the Stats package in the R language R 4.0.3 (R Foundation for Statistical Computing, Vienna, Austria) and the SciPy package in Python (IBM-SPSS, Chicago, IL, USA), with a *p*-value < 0.05, VIP (variable importance in the projection) > 1, and differential expression multiple fold change (FC) > 1.5. The volcano plot showed strong differential expression between Ta and CK (Figure 2C), and a total of 64 DAMs in roses (47 upregulated and 17 downregulated) were induced by *P. pannosa*. The contents of dehydro(11,12) ursolic acid lactone (DUA), betulonic acid, maslinic acid, and trametenolic acid B were significantly upregulated in Ta compared with CK, suggesting that these DAMs could be involved in the responses of roses to *P. pannosa*. The metabolic composition of rose tissue shifted under the influence of *P. pannosa*, resulting in significant alterations in the expression levels of terpene compounds, particularly DUA, maslinic acid, trametenolic acid B, betulonic acid, and ganolucidic acid B. Terpenoids, which are prevalent secondary metabolites in plants, play a crucial role in the management of plant diseases and pests. The names and chemical structures of these five compounds are presented in Figure 3. At the same time, the expression trends and significance of DAMs were analyzed based on the points showing strong differential expression in the volcano plot (Figure 2D). The VIP values of DAMs (DUA, maslinic acid, and trametenolic acid B) were greater than 2. The abundances of these compounds were significantly elevated after induction by powdery mildew. Based on the clustering of DAM expression profiles and VIP value analysis, potential *P. pannosa*-induced defensive metabolites against *S. exigua* were further screened. From the 47 overall upregulated DAMs, 30 key candidate compounds were finally selected by prioritizing terpenoid members with high VIP values, as listed in Table 1. A comprehensive literature review across various databases confirmed that none of the 30 candidate compounds, including the five representative metabolites chosen for further validation, have been previously documented to exhibit anti-*S. exigua* activity.

Nevertheless, the insect-resistance potential of these metabolites has thus far only been inferred from their pathogen-induced expression patterns and chemical properties. To clarify their ecological defensive functions, host selection behavioral assays are necessary to verify their direct effects on the feeding behavior of *S. exigua* larvae.

### 2.3. Antifeedant Activity of Individual Compounds Against S. exigua

The terpenoids exhibiting significantly upregulated expression patterns among the DAMs were selected, and their effects on the feeding of *S. exigua* were evaluated. As shown in Table 2, these compounds exhibited varying degrees of resistance against *S. exigua* at different concentrations. The amount of feeding by the insects on rose leaf discs treated with the compounds was significantly lower than that on the control leaf discs. Specifically, at concentrations of 0.01 mg/mL, 0.05 mg/mL, and 0.1 mg/mL, the selective antifeedant rates for DUA were 47.65%, 56.59%, and 68.04%, respectively. The non-selective antifeedant rates were 64.54%, 73.01%, and 80.99%, respectively. Additionally, trametenolic acid B, betulonic acid, maslinic acid and ganolucidic acid B exhibited antifeedant rates above 50%. According to Table 1, as the concentration of the compound increased, the antifeedant rate against *S. exigua* increased accordingly. This inhibitory effect is evident in both selective and non-selective feeding scenarios. This observation implies that rose plants synthesize terpenoid secondary metabolites in response to powdery mildew infection, which enhances their resistance to *S. exigua*.

### 2.4. Selective Toxicity of Monomeric Compounds Against S. exigua Larvae

Based on a 24 h dual-choice bioassay, Table 3 presents the EC_50_ values of the five monomeric compounds. These values were determined in the following order: ganolucidic acid B (EC_50_ = 0.014 mg/mL), DUA (EC_50_ = 0.015 mg/mL), trametenolic acid B (EC_50_ = 0.067 mg/mL), betulonic acid (EC_50_ = 1.189 mg/mL), and maslinic acid (EC_50_ = 1.256 mg/mL), with a higher EC_50_ value indicating a safer profile. When combining the EC_50_ data from Table 3 with overall efficacy, trametenolic acid B, ganolucidic acid B, and DUA emerge as highly effective agents against *S. exigua* larvae.

## 3. Discussion

Studies have shown that infection with *P. pannosa* stimulates rose plants to produce insecticidally active compounds, thereby enhancing their resistance to *S. exigua*. However, there are limited reports on the application of metabolomics to investigate host plant-mediated interactions between diseases and pests [7]. Among the identified metabolites, ten volatile components—including hexadecanol—exhibited significant repellency against *S. exigua* oviposition. This research provides insights into the chemical mechanisms of powdery mildew-induced resistance in roses against *S. exigua* and emphasizes the role of plant volatile compounds [8]. Nevertheless, the effects of non-volatile metabolites on *S. exigua* remain unclear and require further investigation. In this study, we analyzed rose leaves before and after *P. pannosa* infection using ultra-high-performance liquid chromatography–mass spectrometry (UHPLC-MS) to screen for differentially expressed metabolites. LC-MS metabolomics was used to reveal significant alterations in the metabolic components of roses following *P. pannosa* infection.

According to the results in Figure 1, extracts from mildew-infected roses exhibited antifeedant effects on *S. exigua* larvae, indicating that roses develop resistance to *S. exigua* following *P. pannosa* infection. Previous studies have shown that betulonic acid and its derivatives repel the cutworm moth *Spodoptera litura* in its larval stage [11] and that betulonic acid acts as an antifeedant against the Colorado potato beetle [23]. The compounds ursolic acid lactone (**A**), maslinic acid (**B**), and trametenolic acid B (**C**) had EC_50_ values of 0.015 mg/mL, 1.256 mg/mL, and 0.067 mg/mL, respectively, indicating their effectiveness against *S. exigua*. Among them, DUA exhibited the strongest inhibitory effect, significantly suppressing larval feeding behavior. These results suggest that these triterpenoids may act as chemical signals influencing host selection by *S. exigua*, although their practical efficacy in pest control requires further validation. Nevertheless, DUA, trametenolic acid B, ganolucidic acid B, betulonic acid, and maslinic acid play key roles in plant–insect interactions by mediating the identification of susceptible roses. Their application could avoid environmental pollution and support the development of eco-friendly biopesticides, offering promising biodegradable solutions for pest management in rose cultivation.

Within a particular ternary system, the capacity of insects to detect disease in their host plant is influenced by the specific insect, plant, and pathogen species present. A recent systematic review confirms that herbivorous insects exhibit adaptive oviposition responses to plant disease and stress, but the strength of this preference–performance relationship varies widely depending on the specific insect–host plant combination [24]. Specifically, Jones [24] reported that 50% of insect species adaptively avoided diseased native hosts, with no maladaptive responses observed. This species-specific pattern aligns closely with our finding that *S. exigua* behavior was altered only by *P. pannosa*-infected roses, confirming the system-dependent nature of pathogen-mediated effects. These interactions are commonly mediated by plant primary and secondary metabolites, whose profiles differ across species and pathosystems, thereby shaping distinct behavioral outcomes for the insect [25].

This paper focuses on triterpenoids—widely distributed plant secondary metabolites that play a pivotal role in regulating plant disease and pest resistance. Triterpenoids display various biological activities, including anti-insect and repellent effects against herbivorous insects, such as *Spodoptera exigua*.

For instance, our results demonstrate that five non-volatile triterpenoid metabolites (dehydro(11,12) ursolic acid lactone, maslinic acid, trametenolic acid B, betulonic acid, and ganolucidic acid B) induced by *Podosphaera pannosa* infection in *Rosa chinensis* exhibit significant dose-dependent antifeedant activity against *S. exigua* larvae. Among them, ganolucidic acid B and dehydro(11,12) ursolic acid lactone showed the strongest effects, with EC_50_ values of 0.014 mg/mL and 0.015 mg/mL, respectively.

Previous studies have shown that terpenoid volatiles induced by *S. exigua* feeding can mediate indirect defense by attracting natural enemies [26], highlighting the multifaceted role of triterpenoids in plant resistance to this herbivore. In this study, five triterpenoid compounds—DUA (**A**), maslinic acid (**B**), trametenolic acid B (**C**), betulonic acid (**D**), and ganolucidic acid B (**E**)—were selected based on three criteria. First, metabolomic analysis revealed that these compounds were significantly upregulated (1.53- to 354.19-fold) in powdery mildew-infected roses, serving as key differential metabolites in rose defense against pathogens. Second, all five compounds are triterpenoids, a class of natural defense compounds with proven efficacy against pests and diseases [27,28]. Third, VIP analysis confirmed their pivotal roles in distinguishing metabolic phenotypes between healthy and infected roses, establishing them as core mediators of rose resistance to *S. exigua*. This integrated strategy ensured a strong correlation between the selected compounds and insect resistance, thereby improving the success rate of activity verification and providing precisely targeted candidates for elucidating the chemical mechanism underlying powdery mildew-induced insect resistance in roses.

Triterpenoids are the focus of the present study. Previous studies have confirmed that pentacyclic triterpenoids—particularly the ursane-type ursolic acid lactone and lupane-type betulonic acid identified via LC-MS—exhibit significant insect-repellent activity. These natural plant metabolites are environmentally benign due to their ready biodegradability and low non-target toxicity [29]. Our findings are consistent with a recent study by Liu et al. [30], which demonstrated that reduced triterpenoid levels in phytoplasma-infected jujube leaves were associated with increased feeding by the insect vector *Hishimonus hamatus*. Together, these results support the conclusion that triterpenoids play a critical role in mediating plant–insect interactions.

Following *P. pannosa* infection, rose plants exhibited significant alterations in terpenoid expression levels. Among the 15 compounds with markedly increased expression, some (e.g., medolic acid) did not affect *S. exigua* feeding behavior. In contrast to Yang et al. [7], who examined volatile-mediated resistance of roses to *S. exigua*, the present study investigates non-volatile substances, clarifying the resistance mechanisms underlying *P. pannosa*-induced defense in roses against *S. exigua* from a novel perspective. Notably, some of these compounds are not yet commercially available, underscoring the need for further research on upregulated terpenoids. Although several compounds effectively reduced *S. exigua* feeding, their effects emerged gradually. The triterpenoid-based plant defense compounds identified in this study are environmentally benign and target-specific, showing great potential for use in integrated pest management (IPM) strategies alongside other control methods to achieve sustainable and effective pest control.

The essence of *Podosphaera pannosa*-induced insect resistance in *Rosa chinensis* lies in the fact that pathogen infection activates the triterpenoid biosynthesis pathway in plants, which specifically upregulates five highly bioactive non-volatile triterpenoid metabolites. The five non-volatile triterpenoid metabolites induced by *P. pannosa* infection, namely dehydro(11,12) ursolic acid lactone (DUA), maslinic acid, trametenolic acid B, betulonic acid, and ganolucidic acid B, exert potent antifeedant activity against *Spodoptera exigua*, significantly suppressing pest feeding and thereby enhancing the insect resistance of the host. This mechanism clarifies the core role of non-volatile triterpenoids in the ternary system of *R. chinensis*–*P. pannosa*–*S. exigua*, complements the chemical framework underlying plant-mediated pathogen–insect interactions, and provides valuable molecular targets for the development of environmentally friendly plant-derived pesticides. Notably, DUA showed an extraordinary 354-fold increase, being nearly undetectable in healthy roses. This confirms it as a specific and critical defensive metabolite induced by *P. pannosa*.

As anticipated, the five triterpenoids identified in *P. pannosa*-infected roses exhibited pronounced antifeedant activity against *S. exigua* larvae. Zhang et al. [31] documented that terpenoid alkaloids from Aconitum species exerted potent feeding deterrent effects on *S. exigua*, with the most active compound yielding an EC_50_ of 0.07 mg/cm^2^. In the present study, dehydro(11,12)ursolic acid lactone and ganolucidic acid B demonstrated superior efficacy, with EC_50_ values of 0.014–0.015 mg/mL. Such congruence corroborates that triterpenoids represent a class of effective antifeedants against *S. exigua*, consistent with the established bioactivity profile of terpenoids documented in prior research. Elucidating these mechanisms is crucial not only for advancing our understanding of pest–plant interactions in natural ecosystems but also for developing effective and sustainable IPM strategies.

## 4. Materials and Methods

### 4.1. Plants and Insects

Rose plants of *R*. *chinensis* cv. ‘Movie Star’ were planted in 2015 in commercial cut rose greenhouses in Chenggong County, Yunnan Province, southwestern China. Greenhouse conditions were maintained at approximately 26 °C during the day and 15 °C at night, with a relative humidity of about 70% and 6–8 h of natural light per day. Healthy and *P. pannosa*-infected rose plants at the same growth stage were collected from these greenhouses in mid-July 2024 to minimize experimental error. The symptomatic plants were naturally infected and exhibited typical powdery mildew symptoms. Based on well-documented pathological characteristics and the local epidemiological background, the diseased roses were confirmed to be predominantly infected by *P. pannosa*.

*S. exigua* larvae were collected from the same greenhouse, and their artificial diet was purchased from Henan Jiyuan Baiyun Industrial Co., Ltd. (Jiyuan, China). The larval lines were sensitive lines raised indoors for three generations and had no history of pesticide exposure. These larvae were maintained in a controlled rearing environment at 27 °C with 80% relative humidity and a 14:10 h light/dark cycle until they reached the pupal stage. Initially, the larvae were fed rose leaves; after emerging as moths, they were provided with a 10% (*w*/*v*) honey–water solution. Males and females were reared separately. Subsequently, adult *S. exigua* were transferred to the insectary in a predetermined ratio to facilitate effective mating and oviposition. The honey–water solution and rose leaves in the beakers were regularly replaced to ensure *S. exigua* could replenish their energy promptly. For the experiments, eggs were deposited on rose leaves placed within a fine steel wire cage, and the newly hatched larvae were raised in the same manner as previously described.

### 4.2. Extract Preparation

Healthy and *P. pannosa*-infected rose leaves were cut off and washed with water, then soaked in 50% methanol (analytical grade) for 72 h, with agitation every 24 h for 5 min (repeated three times). At the end of the immersion, the extracts were filtered by suction and concentrated by evaporation. The extracts were diluted with methanol to 5 mg/mL, using a method adapted from Almpounioti et al. [32].

### 4.3. Effect of Extracts on the Feeding Behavior of S. exigua

The three-choice bioassay was conducted using solutions prepared as described during the extract preparation, namely, 5 mg/mL healthy rose leaf extract (CK1), 5 mg/mL *P. pannosa*-infected rose leaf extract (T), and a 50% methanol solution (CK2). Fresh, tender, and uniformly sized rose leaves were selected, cleaned with deionized water, and air-dried. Instead of leaf disks, intact leaf segments were utilized. Each segment was fully submerged in one of the three solutions for 5 s, then removed and air-dried at a distance to prevent cross-contamination. Petri dishes (15 cm in diameter, Sangon Biotech Co., Ltd., Shanghai, China) were pre-cleaned with tap water, followed by deionized water, sterilized, dried, and allowed to cool to room temperature. Labels were affixed externally to divide the inner space into three equal regions marked A-A (CK1), B-B (T), and C-C (CK2). After drying, the leaf segments were placed in their corresponding regions with adaxial surfaces facing upward, and the petioles were wrapped in absorbent cotton (Jiangsu Yabang Medical Supplies Co., Changzhou, China) moistened with sterile water to maintain freshness. Third-instar larvae of *S. exigua*, which had been fed on cabbage leaves after hatching, were selected for uniform body weight and starved for 8 h. Then, one larva was introduced into the center of each dish. Each treatment group comprised 15 dishes with 3 parallel replicates. Twenty-four hours post-treatment, the consumed leaf area was quantified by tracing feeding holes on 1 mm^2^ transparent grid paper (Deli Group Co., Ltd., Ningbo, China) and calculating the total area. The three-choice feeding bioassay was adapted from the protocol described in Nansen et al. [33].

To begin with, *P. pannosa* was inoculated onto healthy rose plants using a spore-suspension inoculation method, as described previously [34]. Six replicates were set up for metabolomic analysis, with 12 samples numbered CK4–CK9 (healthy) and Ta4–Ta9 (*P. pannosa*-inoculated). For each sample, 100 mg of rose leaf tissue was collected and ground into a fine powder with liquid nitrogen. Subsequently, 50 μL of the homogenate was aspirated using a pipette and transferred to a 1.5 mL centrifuge tube. Next, 400 μL of extraction solvent (acetonitrile:methanol = 1:1, *v*/*v*) was added. The mixture was vortexed for 30 s and then subjected to low-temperature ultrasonic extraction using an ultrasonic cleaner (SBL-10TD, Ningbo Scientz Biotechnology Co., Ltd., Ningbo, China) for 30 min (5 °C, 40 kHz). The samples were allowed to stand at −20 °C for 30 min and then centrifuged in refrigerated centrifuge (Model 5430R, Eppendorf AG, Hamburg, Germany) at 4 °C and 13,000× *g* for 15 min. The processed solution was subjected to low-temperature ultrasonic extraction for 5 min (5 °C, 40 KHz) followed by centrifugation at 4 °C and 13,000× *g* for 5 min. The supernatant was transferred to a centrifuge tube with an analytical insert. In addition, to ensure analytical stability, all samples were evenly mixed to prepare quality control (QC) samples. During testing, QC samples were inserted after every three analytical samples.

Untargeted metabolomic analysis was performed on all sample extracts using an ultra-high-performance liquid chromatography–quadrupole time-of-flight mass spectrometry system (UHPLC-Q-TOF-MS; Thermo Fisher Scientific, Q Exactive Plus, Waltham, MA, USA). The samples were separated on an HSS T3 column (100 mm × 2.1 mm internal diameter, 1.8 μm particle size) with an injection volume of 2 μL, followed by detection with a mass spectrometer (Q Exactive HF-X, Thermo Fisher Scientific, Framingham, MA, USA). Mobile phase A consisted of 95% water and 5% acetonitrile (with 0.1% formic acid), whereas mobile phase B comprised 47.5% acetonitrile, 47.5% isopropanol, and 5% water (with 0.1% acetic acid). Gradient elution was performed under the following conditions: from 0 to 0.1 min, mobile phase B was increased linearly from 0% to 5%; from 0.1 to 2 min, from 5% to 25%; from 2 to 9 min, from 25% to 100%; from 9 to 13 min, the mobile phase was maintained at 100%; from 13.0 to 13.1 min, it was decreased linearly from 100% to 0%; and from 13.1 to 16 min, it was held at 0%. The flow rate was set at 0.40 mL/min, and the column temperature was maintained at 40 °C. The mass spectrometry settings were as follows: in positive mode, the ion spray voltage was set to 3500 V, and in negative mode, to 2800 V; the sheath gas flow rate was 40 psi, and the auxiliary heating gas flow rate was 10 psi. The ion source temperature was 400 °C, and the instrument operated with collision energies cycling between 20, 40, and 60 V. The resolution was calibrated to 70,000 for MS1 and 17,500 for MS2. Both positive and negative ion scan modes were used, covering an *m*/*z* range of 70–1050. The analytical conditions were adapted and optimized from a previously reported method [35]. The raw LC-MS data were processed using Progenesis QI (Waters Corporation, Milford, CT, USA) for baseline filtering, peak detection, retention time correction, integration, and peak alignment, resulting in a data matrix that included retention time, mass-to-charge ratio (*m*/*z*), and peak intensity. Furthermore, the MS and MS/MS spectral information was matched against public metabolic databases, HMDB (http://www.hmdb.ca/) and Metlin (https://metlin.scripps.edu/), to acquire comprehensive details about the metabolites. The matrix data obtained from the database were uploaded to the MajorBio Cloud Platform (https://cloud.majorbio.com) for subsequent analysis. To minimize the impact of data variations and optimize the screening of differentially accumulated metabolites (DAMs), preprocessing was conducted initially. The original dataset, which included both quality control and test samples, underwent a series of systematic preprocessing steps: missing value filtering, data imputation, standardization, quality control validation, and data conversion.

The R package ropls (version 1.6.2) was used to perform principal component analysis (PCA). Seven-fold cross-validation was employed to assess the stability of the model. Peak alignment was performed using Progenesis QI 2.3 software, and volcano plots were generated using the ggplot2 package in R. The screening criteria for differential metabolites were VIP > 1 (calculated by the OPLS-DA model).

### 4.4. Determination of the Antifeedant Activity of Individual Compounds Against S. exigua

Using a three-stage approach—metabolome difference screening, activity potential prediction, and biological test verification—we sequentially identified five triterpenoid compounds as active agents against *S. exigua*. Each compound was precisely weighed, fully dissolved in methanol, and diluted to a fixed volume to obtain a homogeneous solution. The experimental procedure was identical to that described previously. Third-instar *S. exigua* larvae were placed in incubators, with each treatment group consisting of 15 larvae and six biological replicates. After 24 h of treatment, the leaf area consumed in each group was measured using a nine-grid culture plate and calculated according to the following formula:

(1) $$\mathrm{Antixenosis}\;\mathrm{rate}\;(\%)\;=\frac{\left(CK-T\right)}{\left(CK+T\right)}\times 100\%$$

(2) $$\mathrm{Non}\text{-}\mathrm{antixenosis}\;\mathrm{rate}\;(\%)=\frac{\left(CK-T\right)}{CK}\times 100\%$$

where CK is the feeding area of the healthy rose extract, and T is the feeding area of the powdery mildew-infected extract.

A positive antifeedant rate indicates that powdery mildew infection on roses inhibits the feeding behavior of *S. exigua.* Conversely, a negative antifeedant rate suggests that this infection attracts *S. exigua* feeding.

SPSS 21.0 (IBM SPSS, Chicago, IL, USA) was used to perform statistical analysis on the results from the bioassays of oviposition and feeding behavior of *S. exigua,* and an independent samples Student’s *t*-test was used to determine significant differences.

### 4.5. Determination of the EC_50_ Values

Using *S. exigua* as the research subject, rose compounds induced by *P. pannosa* were selected. Gradient concentrations were established based on variations in compound activity observed in preliminary experiments. Highly active compounds dehydro(11,12) ursolic acid lactone (DUA) and ganolucidic acid B were tested at 0.01–0.1 mg/mL, while moderately to lowly active compounds such as maslinic acid and betulonic acid were tested at 1.0–2.0 mg/mL. Trametenolic acid B was tested at concentrations ranging from 0.05 to 0.25 mg/mL. Blank controls consisted of leaf discs treated with a 50% methanol aqueous solution. A dual-choice bioassay was conducted by placing third-instar *S. exigua* larvae, pre-starved for 8 h and with uniform body weight, in Petri dishes containing both control leaf discs and leaf discs treated with different compound concentrations. After 24 h, larval feeding areas were measured using transparent grid paper. Subsequently, SPSS 21.0 (IBM SPSS, Chicago, IL, USA) software was employed to construct a linear regression model of the compound concentration–antifeedant rate relationship. The fit was validated using R^2^, with a requirement of ≥0.85 (with values for most compounds exceeding 0.9). By substituting an antifeedant rate of 50% into the regression equation, the corresponding concentration, EC_50_ (half-maximal effective concentration), was obtained through inverse calculation, as described previously [31]. Additionally, the 95% confidence interval was computed to ensure the reliability of the results, ultimately quantifying the insecticidal activity of various compounds against *S. exigua*.

### 4.6. Chemicals and Reagents

Deionized water was used throughout the experiments. Methanol, formic acid, acetonitrile, and isopropanol (all HPLC grade) were purchased from Fisher Scientific (Waltham, MA, USA). Ethanol (analytical grade) was also obtained from Fisher Scientific (USA). Formaldehyde was purchased from Shantou Dahao Fine Chemicals Co., Ltd. (Shantou, China). Ursolic acid lactone was obtained from Bailingwei Technology Co., Ltd. (Beijing, China). Maslinic acid, betulonic acid, and trametenolic acid B were purchased from Wuhan Tianzhi Biological Technology Co., Ltd. (Wuhan, China). Ganoderic acid B was obtained from Yunnan Xili Biological Technology Co., Ltd. (Kunming, China). All other chemicals and solvents used in this study were of analytical grade and were commercially available.

## 5. Conclusions

This study explored the chemical mechanism responsible for *Podosphaera pannosa*-induced resistance in *Rosa chinensis* against *Spodoptera exigua*. Analysis using untargeted metabolomics demonstrated a substantial modification in the metabolomic profiles of rose leaves upon *P. pannosa* infection, revealing a total of 64 differentially accumulated metabolites (DAMs). Notably, five non-volatile triterpenoid metabolites exhibited significant upregulation: dehydro(11,12) ursolic acid lactone (DUA), maslinic acid, trametenolic acid B, betulonic acid, and ganolucidic acid B.

Antifeedant bioassays revealed that all five triterpenoids exhibited significant dose-dependent antifeedant activity against third-instar *S. exigua* larvae. Ganolucidic acid B (EC_50_ = 0.014 mg/mL) and DUA (EC_50_ = 0.015 mg/mL) demonstrated the highest bioactivity, with non-selective antifeedant rates of 79.82% and 80.99%, respectively, at a concentration of 0.1 mg/mL. These results suggest that *P. pannosa* infection enhances the resistance of host plants by activating the triterpenoid biosynthesis pathway in roses. This activation specifically upregulates the accumulation of these bioactive non-volatile triterpenoids, thereby significantly suppressing larval feeding behavior and enhancing host plant resistance.

## Figures and Tables

**Table 1:** Top 30 *P. pannosa*-induced DAMs of roses.

No.	Metabolite	Regulated	*m*/*z*	FC (TA/CK)	*p*_Value
1	Dehydro(11,12) ursolic acid lactone	up	455.3507	354.1869	0.00000
2	Tomentosic acid	up	487.3406	3.6644	0.00031
3	Trametenolic acid B	up	457.3672	3.3272	0.00003
4	Capsianoside VI	up	961.4408	2.3678	0.02853
5	Acoric acid	up	581.3329	2.2669	0.00081
6	Vaccinoside	up	501.1344	2.0071	0.00519
7	Ganolucidic acid B	up	547.3286	1.8659	0.00081
8	16-Acetylpriverogenin A	up	515.372	1.8449	0.00007
9	Maslinic acid	up	473.3617	1.7219	0.00000
10	Suspensolide F	up	523.2038	1.6132	0.00046
11	Perilloside C	up	339.1802	1.5772	0.00073
12	Lucyoside Q	up	679.4091	1.5597	0.00001
13	Betulonic acid	up	455.3516	1.5367	0.00000
14	Cinnamoside	up	563.2362	1.5342	0.02696
15	Momordicoside K	up	631.4212	1.3715	0.00031
16	Medicagenic acid	up	547.3282	1.3402	0.00029
17	Glyyunnansapogenin B	up	489.3568	1.2927	0.00032
18	Phytolaccoside A	up	693.3873	1.251	0.00171
19	Glucosyl passiflorate	up	695.4038	1.2467	0.00019
20	Theasapogenol E	up	505.35	1.238	0.01499
21	Trans-p-Menthane-7,8-diol 7-glucoside	up	379.1969	1.227	0.00337
22	Quassinol	up	361.1646	1.2149	0.0004
23	Icariside B8	up	371.2065	1.1884	0.00019
24	Secoeremopetasitolide A	up	331.1542	1.184	0.001
25	Ligustroside	up	525.1958	1.1659	0.00057
26	Ginsenoside Rh8	up	675.3843	1.1469	0.04296
27	Goshonoside F3	up	609.3257	1.1373	0.00272
28	Ziziphin	up	979.5183	1.1254	0.00015
29	Tsangane L 3-glucoside	up	375.2378	1.1181	0.00029
30	Dehydrosaponin I	up	961.4734	1.092	0.00124

*m*/*z* (mass-to-charge ratio) refers to the ratio of the mass of charged ions to the charge.

**Table 2:** Determination of the feeding behavior of *S. exigua* by five compounds.

PA	Concentration (mg/mL)	Average Area Eaten in mm^2^		Antifeedant Rate/%	
		Control	Treatment	Selective	Non-Selective
**A**	0.01	62.07 ± 11.02 a	22.00 ± 4.32 b	47.64	64.54
	0.05	78.41 ± 18.65 a	21.10 ± 7.04 b	56.59	73.01
	0.1	87.15 ± 19.96 a	16.57 ± 5.29 b	68.05	80.99
**B**	1	84.41 ± 23.46 a	33.95 ± 6.37 b	42.64	59.78
	1.5	76.75 ± 23.69 a	22.97 ± 7.24 b	53.94	70.08
	2	43.81 ± 8.79 a	8.23 ± 2.26 b	68.36	81.21
**C**	0.05	29.80 ± 5.55 a	10.43 ± 2.85 b	48.14	64.98
	0.1	33.67 ± 7.12 a	10.48 ± 2.63 b	52.52	68.87
	0.25	47.61 ± 10.48 a	11.76 ± 3.20 b	60.39	75.3
**D**	1	55.01 ± 11.86 a	19.15 ± 5.30 b	48.35	65.18
	1.5	39.40 ± 9.93 a	12.34 ± 2.11 b	52.3	68.68
	2	51.20 ± 9.64 a	15.03 ± 4.41 b	54.62	70.65
**E**	0.01	23.75 ± 6.93 a	8.18 ± 2.56 b	48.76	65.55
	0.05	29.08 ± 7.20 a	8.25 ± 2.88 b	55.79	71.62
	0.1	41.81 ± 13.79 a	8.44 ± 2.98 b	66.41	79.82

Control: Leaf area of *S. exigua* feeding on deionized water treatment. Treatment: *S. exigua* fed on the leaf area treated with the tested drug. Significant differences are indicated by “a” and “b” (*p* < 0.05), and non-significant differences are indicated by “a”. The identified compounds are DUA (**A**), maslinic acid (**B**), trametenolic acid B (**C**), betulonic acid (**D**), and ganolucidic acid B (**E**).

**Table 3:** Selective antifeedant toxicity of individual compounds against third-instar larvae of *S. exigua*.

Compounds	Y = ax + b	R2	EC50 (mg/mL)	Confidence Interval 95%
**A**	Y = 1.47 + 0.8x	0.934	0.015	0.001~0.029
**B**	Y = −0.34 + 3.48x	0.967	1.256	0.963~1.468
**C**	Y = 0.84 + 0.71x	0.992	0.067	0.013~0.253
**D**	Y = −0.06 + 0.84x	0.997	1.189	0.125~1.676
**E**	Y = 1.25 + 0.67x	0.878	0.014	0.000~0.031

The identified compounds are DUA (**A**), maslinic acid (**B**), trametenolic acid B (**C**), betulonic acid (**D**), and ganolucidic acid B (**E**).

## Data Availability

The original contributions presented in this study are included in the article. Further inquiries can be directed to the corresponding authors.
